# Commercialized “Smudge Sticks” Used as Incense in the Netherlands: An Inventory of Plants and Trends Behind a New Age Fashion

**DOI:** 10.3390/plants13213003

**Published:** 2024-10-27

**Authors:** Isabela Pombo Geertsma, Berber E. Zandstra, Anastasia Stefanaki, Tinde R. van Andel

**Affiliations:** 1 Quantitative Biodiversity Dynamics, Utrecht University Botanic Gardens, Utrecht University, 3508 TD Utrecht, The Netherlands; a.stefanaki@uu.nl; 2 Naturalis Biodiversity Center, P.O. Box 9517, 2300 RA Leiden, The Netherlands; tinde.vanandel@naturalis.nl; 3 Biosystematics Group, Wageningen University & Research, Droevendaalsesteeg 1, 6708 PB Wageningen, The Netherlands; berber.zandstra@wur.nl

**Keywords:** California, cultural appropriation, Ethnobotany, Europe, Native American, Neo Paganism, ritual plants, United States, Wicca, witchcraft

## Abstract

Incense is essential in religious ceremonies, even in relatively new religious and spiritual movements such as New Age and Neopaganism. These garner little attention from ethnobotanists, although they trigger an international trade in wild-harvested plants. In this paper, we studied the botanical ingredients of smudge sticks (dried plant bundles burned for purification) in the Netherlands, and people’s motivations to use them posing the following questions: what plant species are included in smudge sticks? what are they used for? and are exotic plants preferred over native Dutch plant species? We visited online and physical shops in Dutch cities, acquiring a total of 29 different smudge sticks containing at least 15 species. We held semi-structured interviews with 11 users, vendors, and herbal experts, and collected data from 33 questionnaires. *Salvia apiana* L. was most frequently found, along with North American species of the genus *Artemisia*. The rise of the New Age movement resulted in North American ritual plant species being easily available in (online) shops in the Netherlands and smudge sticks being used for personal protection and cleansing. Despite the smudge sticks’ commercial demand, there is no data regarding the pressure on wild populations of species used in these bundles. For the preservation of these species it is crucial that scientific monitoring of their harvest is undertaken in the future.

## 1. Introduction

The smoke of incense has been an essential component in sacred religious ceremonies for millennia [1,2,3]. Incense is also used in relatively new movements such as New Age and Neopaganism, including Wicca, and resulting popular religions [2,4,5,6,7,8]. Although in the last few decades a growing body of literature was published on these movements, in fields such as history and sociology of religion, and anthropology (e.g., [9,10,11]), from the viewpoint of ethnobotany, New Age, Neopaganism, and contemporary Western popular religions are virtually unstudied. Yet, these relatively new beliefs influence the harvest and trade of wild plants (e.g., [12]) making them a fascinating case for ethnobotanical investigation.

Incense is made of dried plant material, usually processed as incense sticks, granules (usually tree resins), or bound bundles of dried plant leaves and stems. The smoke produced by fire and incense is used ritually to connect with entities or “energies”, to create a certain sacred atmosphere, and for ritual healing by peoples all over the globe. In southwestern China, ritual fumigation is currently practiced to communicate with ancestors, deities and spirits [13]. In Brazil, fumigation is performed in healing ceremonies, among others by practitioners of the Umbanda religion [14]. In Native North American cultures, there are several documented instances of ritual and healing uses of incense [15] (naeb.brit.org, accessed 2 May 2024). Incense smoke is not only used in a religious or ritual context, but also purely as medicine against, for example, respiratory tract and skin diseases [13,16], for repelling house flies [17], disguising bad smells [7], and even measuring time based on how long an incense stick takes to burn completely [18]. These examples just scratch the surface of a wide and vast variety of incense usage in the world [3]. 

Although incense, especially frankincense (*Boswellia* spp.), has been ceremonially used in the Catholic church for centuries [19,20], including in the Netherlands where this study was conducted, a new and secular market has emerged for smudge sticks made from dried plant bundles. Their packaging and sales pitch typically associate them with Native North American spirituality. Tightly bound dried plant bundles are indeed used by some Native North American cultural groups in traditional ceremonies [2,15] (naeb.brit.org, accessed 2 May 2024). They are ignited on one end to create smoke which, depending on the area or people, is intended as medicine, to purify spaces and/or people, to keep danger at bay, to provide a nice smell, and to deter insects [2,21,22] (naeb.brit.org, accessed 2 May 2024). In the United States of America (USA), examples of plant species employed for smudging in the Native American Ethnobotany Database (naeb.brit.org, accessed 29 July 2024) are sweetgrass or vanillagrass (*Hierochloe odorata* (L.) P.Beauv.), various species of *Artemisia* and *Salvia apiana* Jepson, grouped under the name ‘sage’, Eastern Arborvitae (*Thuja occidentalis* L.), and juniper (*Juniperus* spp.), depending on the cultural groups and geographical area. The local terms applied to these plant bundles differ per group, but they are generally referred to as incense or, more commonly, smudge sticks in (popular) literature.

Indigenous smudge sticks were adopted for ceremonial purposes in the USA by practitioners of New Age [23], Paganism [2,24,25], and Wicca [26,27,28] in the 20th century. The definitions and contours of these (religious) movements are debated (e.g., [6,10,11,29]). Notably, they are growing in the Netherlands [30]. Especially New Age and to a lesser extent Neopaganism commodify rituals and associated objects from other religions and cultures, not only smudge sticks, but also drums, precious stones, and dreamcatchers [23,31]. This use of ritual objects outside their original native context sparked controversy and is recurrently termed cultural appropriation [23,32,33,34]. There are indications that some plants traditionally used for smudging, such as white sage (*Salvia apiana*), are unsustainably harvested to meet the growing demand in Western countries [35]. However, academic literature mentioning plant use by New Age or Neopagan practitioners during ceremonies is hardly backed up by herbarium specimens, photographs of plants, or plant identifications during fieldwork by botanists (e.g., [23,24,36]). Additionally, so far no research has been carried out on species used in smudge sticks and their uses. 

The first aim of this paper is to identify the diversity of botanical ingredients in commercialized smudge sticks in the Netherlands. Our second aim was to determine why people choose certain species for smudging. We posed the following questions: (1) what plant species are included in smudge sticks in the Netherlands, (2) for what reason are they included, and (3) are exotic plants preferred over native Dutch species, and why? To answer these questions, we collected smudge sticks from shops to identify the species and interviewed users, vendors and herbal experts. We argue that the rise and commercialization of the New Age movement in the 1980’s, its idealization of Native North American spirituality favoring certain smudge stick plant species, and the subsequent easy availability of smudge sticks in shops in the Netherlands, and beyond, led to their growing popularity and thus demand. This is possibly causing the unsustainable harvest of wild species for smudge sticks, which should compel conservationists to develop conservation strategies to protect the species’ populations resilience.

## 2. Results

### 2.1. Botanical Ingredients of Smudge Sticks Sold in Dutch (Web) Shops

**Figure 1:**
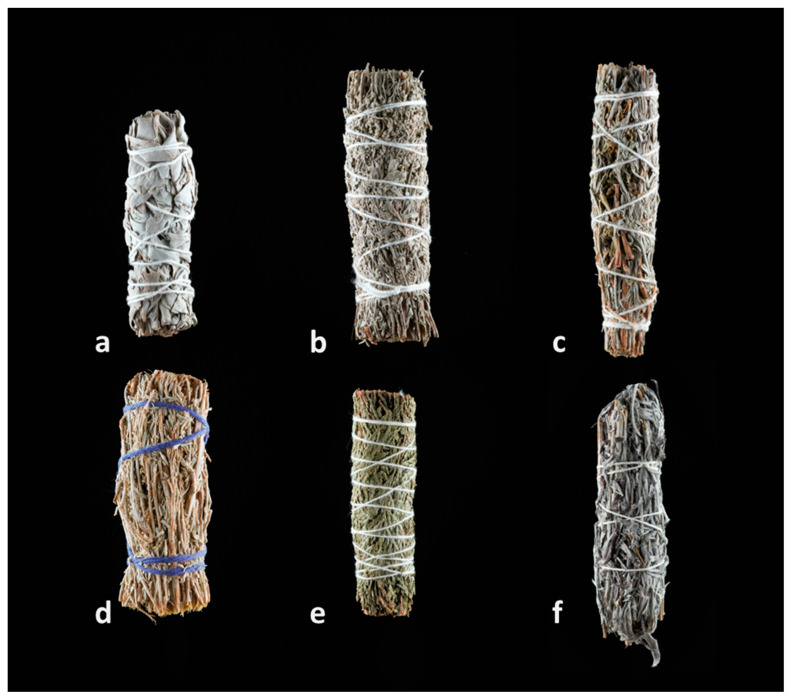
Collage of collected smudge sticks sourced from (online) shops in the Netherlands. (**a**) *Salvia apiana*; (**b**) *Artemisia tridentata*; (**c**) *A. ludoviciana* and *Calocedrus decurrens*; (**d**) *A. tridentata*; (**e**) *Thuja* sp.; (**f**) *A. ludoviciana*. Pictures by I. Pombo Geertsma and C. van der Linden.

**Figure 2:**
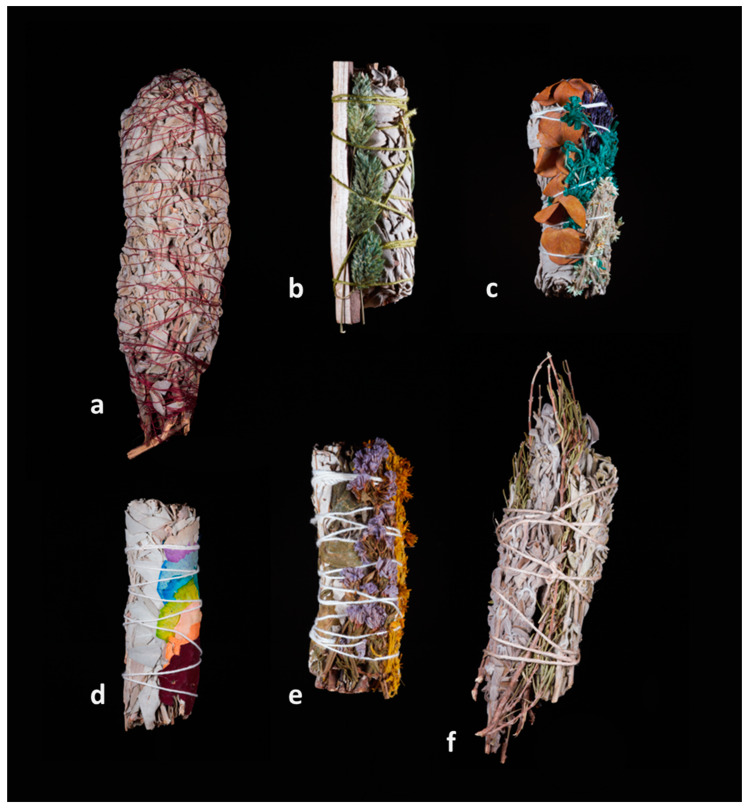
Collage of partly colored smudge sticks composed of *Salvia apiana* mixed with other species, and smudge sticks composed of only European species. (**a**) *S. fruticosa;* (**b**) *S. apiana*, *Phalaris* sp., and *Anacardiaceae* sp.; (**c**) *S. apiana*, *Eucalyptus* sp., cf. *Pseudognaphalium* sp., and one unidentified plant. (**d**) *S. apiana* with *Rosa* sp. petals; (**e**) *Eriodictyon* sp., *Limonium sinuatum*, and cf. *Pseudognaphalium* sp.; (**f**) *S. officinalis* and *S. rosmarinus*. Pictures by I. Pombo Geertsma and C. van der Linden.

In the Netherlands, smudge sticks are found in (web)shops that specialize in spiritual and esoteric products, but also in (herbal) tea shops with an ‘esoteric product corner’. Such products are imported in the Netherlands without restrictive regulations unless they contain CITES species, in which case a phytosanitary permit is necessary (https://www.nvwa.nl/onderwerpen/op-reis-welke-planten-dieren-en-producten-mogen-mee/ik-wil-planten-of-plantaardige-producten-meenemen-naar-nederland, accessed on 7 October 2024). We collected a total of 29 smudge sticks from the online and physical shops, some of them containing several species (Figure 1 and Figure 2). In total, we identified 14 genera in 12 families. Of these, we were able to identify 15 species, but 27 plant ingredients were not identifiable to species level because they missed crucial morphological characteristics (Table 1). In particular, the fragments of *Artemisia* subg. *Tridentatae*, cf. *Pseudognaphalium* and *Eriodictyon* lacked sufficient flower and fruit material and leaf characteristics for species identification. The Asteraceae family was the most represented with at least six species, of which there were at least four in the *Artemisia* genus (*A. californica*, *A. ludoviciana, A. tridentata*, and *A. vulgaris*), one or two cf. *Pseudognaphalium* species, and *Matricaria chamomilla*, followed by Lamiaceae with four *Salvia* species (*S. apiana*, *S. fruticosa*, *S. officinalis*, and *S. rosmarinus*). Most species that we found in the smudge sticks have their natural distribution range in the USA and were bought from North American wholesalers. *Bursera graveolens* was the only solely woody stick that we found, and has a Middle to South American distribution range [37]. One vendor made the smudge sticks herself with wild or cultivated plant material collected around her town. The species used were *Artemisia vulgaris*, *M. chamomilla*, *Hypericum perforatum*, *S. officinalis*, *Rosa* sp., and *Verbena bonariensis*. One couple grew *A. ludoviciana*, which has its natural distribution in North America, on a small commercial scale and made smudge sticks out of this species to sell to an online shop. Of the collected smudge sticks, ten were made of or included leaves and stalks of white sage (*S. apiana*), which has its natural range in California. Fourteen smudge sticks were mixed bundles of two or more species, sometimes they had different geographic origins and cultural traditions, such as *S. apiana* (USA) combined with *Eucalyptus* sp. leaves, which has an Australian origin but is cultivated world-wide; and *S. apiana* with colored rose petals (*Rosa* sp.) referring to chakras from Hinduism and Buddhism. We were unable to identify one plant specimen because it was painted and too few morphological characters could be recognized (dark blue in Figure 2c). 

**Figure 3:**
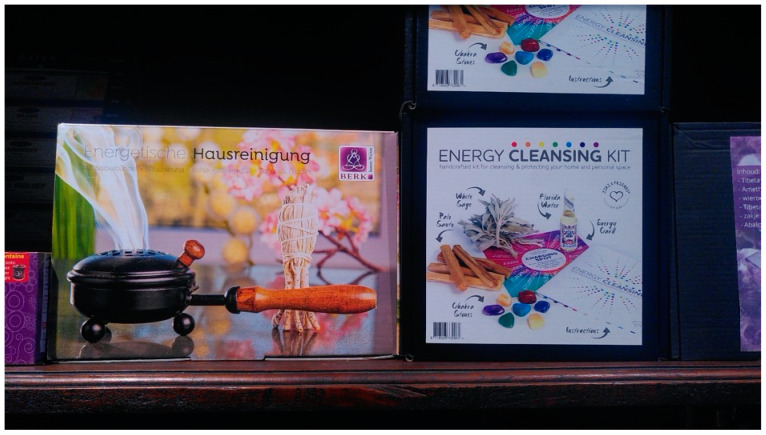
Energy cleansing packages on sale in a shop in Utrecht, March 2023. To the left, a package is sold combining a white sage smudge stick with an iron pan, and to the right, an energy cleansing kit combining white sage (probably *S. apiana*) with palo santo (possibly *Bursera graveolens*), Florida water, colorful gemstones (“chakra stones”), an energy card and an instructions booklet. Picture by I. Pombo Geertsma.

We found smudge sticks or loose white sage leaves added to “cleansing kits” (Figure 3). Some vendors only included Dutch, wild or cultivated, plant species in their smudge sticks, such as *A. vulgaris*, *S. officinalis*, and *M. chamomilla*. One significantly larger smudge stick bought in 2023 contained *S. fruticosa* (Figure 2a) and was sold as “Greek Ceremonial Sage”. The picture on the packaging placed it on an abalone shell that is normally associated with *S. apiana*. The Dutch text on the box was translated as: “*This wild-picked sage from the mountains of Greece has traditionally been used to purify and smudge spaces. In contrast to the North American variant, this sage is much softer. The scent is fresh, feminine, and with a hint of lemon. Partly due to the slow drying process in the Greek sun, the scent and strength of this sage is exceptional.*” 

One of our collected smudge sticks contained a woody stick that was incorrectly labeled as *Pistacia lentiscus* (Figure 2b). Although we did find out that this identification was incorrect, we were unable to identify it further than the family level (Anacardiaceae) due to the challenge in finding out where the species originated from, as wholesalers did not respond, answered that they could not disclose that information, or simply said that they did not know. 

### 2.2. Species and Uses Mentioned in Interviews and Questionnaires

Only two species associated with smudge sticks were mentioned by all four interviewees who were active in the herbal industry: bijvoet (*Artemisia vulgaris*) and lavendel (can refer to several cultivated *Lavandula* species). For lavendel, *L. dentata* was identified in commercialized smudge sticks, and also *A. vulgaris*. The reasons for smudging these species were diverse and differed among the interviewees. Reasons for burning *A. vulgaris* included: to prepare for a workshop, it is easily found, the smell is nice, to gain creative thoughts, to bring a good “vibe” into the house, to cleanse, against angry spirits, and for improving the atmosphere. Reasons for burning *Lavandula* were to cleanse, its calming properties, for love rituals, to protect children, its nice smell, and its protective and cleansing ability ‘on another level’ compared to other smudge sticks.

In the 33 questionnaires at the witches’ fair, 22 people mentioned the use of sage (“salie”, likely *Salvia* spp.), of which three people specifically wrote white sage (“witte salie” presumably *S. apiana*, but they could also mean *S. officinalis*). White sage and sage were most often (17 people) used for purification (sometimes of the house) while two people mentioned protection. Others did not clarify its use or wrote smoking, disinfecting, cooking, ritual, and spiritual as uses for (white) sage. Two other species were also mentioned specifically for ritual incense: *A. vulgaris* (bijvoet) and engelwortel (most likely *Angelica archangelica* L. or possibly *A*. *sylvestris* L.).

### 2.3. Reasons for White Sage Preference

During our semi-structured interviews, when North American plant species were mentioned, we specifically asked why North American species were preferred over species easily available in the wild in the Netherlands. The following explanations were given: people don’t know that you can use native plants for the same purposes; exotic plants work better; native [Dutch] species are boring; white sage is more easily obtainable than native Dutch plants; white sage works better (than native Dutch plants); people don’t even know where the plants are from; people don’t know the difference between native and exotic plants; it’s marketing; white sage smells better; people forgot traditional species; people don’t know that there is a difference between exotic and native species.

Some answers revealed knowledge of the Native American origin of white sage: because it is an American tradition, so you should use white sage; there is an ideal image of the Native North American peoples and to come closer to this ideal people incorporate some of their cultural elements into their own culture; it is from Native Americans, so it should be amazing; it is not a Dutch tradition (at least, not to our knowledge), so no Dutch species are used for this; smudging has to be done with white sage. 

## 3. Discussion

### 3.1. Sustainability of Smudge Sticks 

Our results show that multiple species are used in smudge sticks, of which a considerable portion originate from the USA, specifically western USA, *Salvia apiana* being the most prevalent one in shops. Unlike the other species that we identified, *S. apiana* was encountered in different forms (sticks, loose leaves, and “torches”) and in mixtures with other species. In the wild, it is found in North America in the same ecosystems as *Artemisia californica* [38], which was also found in our smudge sticks. These types of vegetation, coastal sage scrub and chaparral, are known to be threatened by anthropogenic activities such as urban construction and the establishment of agricultural fields [38]. Other taxa that we collected, such as the difficult to identify *Artemisia* subg. *Tridentatae*, cf. *Pseudognaphalium*, and *Eriodictyon* species, are perhaps found in the wild in the same areas and possibly collected at the same time as *S. apiana*. 

Although previous research has exposed that the (commercial) demand for ritual plants may lead to taboos and Indigenous nature conservation strategies [39,40], in the case of white sage there are signals that it is being overharvested in the wild to supply its increased demand [12,41,42,43,44] (https://www.gabrieleno-nsn.us (accessed on 21 October 2024)). These news articles have drawn attention to a possible decline in wild white sage populations, negatively affecting both the wild populations of the species and the peoples that depend on the species for ceremonial purposes. Overharvesting of important ceremonial plant species for commercialization is documented for the small cactus species *Lophophora williamsii* in the USA and Mexico negatively impacting the species’ population resilience and its availability for traditional medicine [45]. It is unknown whether the other North American species found in smudge sticks and the *Bursera graveolens* wooden sticks that probably originate from Middle and/or South America, suffer due to a similar growing commercial demand for ritual plants [24]. Although no ecological research has been done on the effects of commercial wild-harvesting of smudge stick ingredients on wild plant populations, the popularity of *S. apiana* (and possibly to a lesser extent *A. californica*) might pose an extra risk for the resilience of local wild populations, adding significant pressures on the availability of this preferred wild plant species. 

Some of our interviewees were concerned about the potential overharvesting of the species from their natural wild populations. One of them, a keen gardener, managed to grow a collection of *S. apiana* on his balcony. He gathered seeds to grow new ones which he handed for free to anyone wanting their own “sustainable” supply of white sage for personal ritual use. Furthermore, to cater to the demand for locally harvested smudge stick plants, *A. ludoviciana* is grown commercially on a small scale in the Netherlands and sold to a smudge stick vendor. One vendor stated awareness of the potential overharvesting and illegal harvesting of white sage and palo santo although they still chose to sell it due to its demand by customers. Other exotic smudge stick species (e.g., *Eriodictyon* sp., *A. tridentata*, and *A. ludoviciana*) have a wider distribution range (Table 1), are probably in less demand, and there are no signs of a potential decline in species populations due to overharvesting.

The smudge sticks themselves are also often mixes of plants from all over the world. For example, palo santo (Middle and South America) combined with white sage; *Eucalyptus* (Australia) combined with white sage; palo santo combined with an abalone shell; or white sage combined with colored rose petals probably representing the chakra’s (a concept originating from Hinduism and Buddhism). Such combinations seem to reflect an uncritical appropriation of traditions from all over the world (see Section 3.4). Also, these species may indicate a declining supply or increasing price of *S. apiana* on the world market. This might also be the reason behind the commercial presence of Greek ceremonial sage (*S. fruticosa*), which we first noticed in shops in the beginning of 2023. 

Unfortunately we were unable to identify a number of plant fragments to the species level. This was especially the case for *Artemisia* species in the subgenus *Tridentatae* and species in the genus *Eriodictyon*. Our specimens were often highly fragmented and lacked morphological characteristics. For a full assessment of species commercialized for smudge sticks and the sustainability issues these species may face, we suggest collecting plant specimens together with the smudge stick plant gatherers themselves in the USA.

### 3.2. Non-American Smudge Stick Ingredients

A native Dutch plant that came up during the in-depth interviews and the questionnaires and is commercialized to a small extent is *A. vulgaris*, clearly the preferred native Dutch herb for smudging. Perhaps this preference stems from the fact that it was sometimes labeled as “black sage” (not to be confused with *S. mellifera*), linking it to white sage (*S. apiana*), and in this way associating it with smudging. Commonly known in Dutch as bijvoet, *A. vulgaris* is a widespread and very common weed in Dutch pioneer vegetation, and easily identifiable. It was historically used for smudging to protect against evil and lightning strikes in the southern parts of the Netherlands [46]. However, similar uses are known for many other native Dutch plants that are likewise abundant and easily recognizable, such as boerenwormkruid (*Tanacetum vulgare* L.,) [46], but do not appear in smudge sticks. Possibly, the custom of burning *A. vulgaris* entered the New Age movements via moxa therapy, a type of acupuncture where this species is burned. Moxa therapy is part of the medicinal corpus of Traditional Chinese Herbal Medicine and is also performed in the Netherlands [47,48]. However, *A. vulgaris* for smudging was only found in the form of dried plant bundles, not as moxa sticks or moxa powder, although these may also be used by New Age practitioners. 

Another noteworthy example of a non-American ingredient is *S. fruticosa*. Smudge sticks composed of this species are marketed as the feminine variant of the white sage smudge stick. *Salvia fruticosa* is a Mediterranean lowland species, yet it is advertised as originating from wild-harvested individuals from the mountains of Greece, displaying a careless attitude by the wholesalers towards a correct description of the species in question. Moreover, the label claims that this sage “has traditionally been used to purify and smudge spaces”. In the eastern Mediterranean, *S. fruticosa* is known as an ingredient in (medicinal) teas and for the production of essential oils [49,50], but we did not find records of its use in burning rituals, making it plausible that this was made up to diversify the market of smudge sticks. Although smell is the most important indicator for selecting incense species [13], in this case *S. fruticosa* may have also been chosen to substitute *S. apiana* due to morphological resemblance, as both species are covered by dense white indumentum, a common adaptation of plants growing in Mediterranean-type habitats. 

### 3.3. Forgotten Knowledge on Native Plants

As knowledge about common plant species in industrialized countries like the Netherlands is relatively low in laypeople [51,52], so is traditional knowledge associated with these species [53]. Thus, consumers tend to turn towards (web)shops believing that they have done the correct species selection for them and provide them with the “knowledge” of their use. This was confirmed by one of our herbalist interviewees, who, in response to the question of why people prefer exotic plants, answered that “people forgot traditional species” and “it is not a Dutch tradition to burn plants” (as incense). 

Interestingly, in the southern Netherlands fumigation rituals exist in which plants are burned for protection. There, wild and cultivated plants are picked each year and formed into bouquets, blessed in the church on special Roman Catholic dates, dried and kept to burn for protection against evil and thunder if needed. This custom was more widespread across Europe in the past [46] and has been extensively described for Poland [54,55]. Remarkably few people know about this ritual in the rest of the Netherlands, but two of our interviewees (both herbal experts) were aware of it. Its connection to the Catholic church might be a throw-off, assuming that most New Age and Neopagan adherents have more secular backgrounds, but according to Jespers [6] people in the folk religion domain that were influenced by New Age are often also Catholic, so there may have been some influence of these blessed herbal bouquets on the smudge stick species and their popularity. This would need further investigation.

### 3.4. Preference for North American Species in Smudge Sticks and the New Age Movement

From our interviews on smudge sticks and our collection of specimens from shops, we noticed an interplay between the perceived loss of traditional knowledge associated with Dutch plant species (see section above), romanticized ideas about Native Americans, and the wide availability of smudge sticks in (online) shops. The supply of products in New Age shops seems to drive people’s choice of ritual plants alongside online blogs, and social media posts, affecting peoples’ prior knowledge on types of smudge sticks. This is illustrated in remarks such as “it’s marketing” and the fact that only two interviewees reported going out to search for potentially useful plants in nature, although this was not a specific question in our interviews. Smudge sticks were probably introduced through the commercialization of the New Age movement, although none of the interviewees remembered when smudge sticks exactly started appearing on the Dutch market. 

Ideas about Native Americans were visible in some participants’ answers like “North American plants work better than native ones.” The admiration of the natural and Indigenous world is characteristic of New Age and related movements [23,56,57]. This does not only apply to North American Indigenous peoples, but also, for example, Indigenous South African peoples. Although we did not find this specific smudge stick in the Netherlands, in South Africa a native *Helichrysum* sp. is commercialized for smudging (marketed as “South African Sage Smudge Stick”) and labeled to be useful for cleansing, protection, and to connect with “your spirit, guides, and angels” (https://www.michakra.co.za/products/imphepo-smudge-stick, accessed on 31 July 2024). Several species in this genus were traditionally used as incense to connect with ancestors, for protection, to drive away sickness, and as medicine [58]. This idealization has its roots in Romanticism, an 18th-19th century philosophical, literary, and art movement that idealized the natural and Indigenous world and had a huge impact on current Western thought and significantly influenced New Age and affiliated movements. Although New Age has its origins in Western society, elements from other cultures are transformed and squeezed into its framework [9]. The movement developed from the 1950s onwards, and from the 1980s it started becoming commercialized [9]. This commercialization incorporated and fused elements and symbols from countless religions or world-view systems, such as yoga, ayurveda, Tibetan Buddhism, and various Aboriginal Australian [59] and Native North-American [33] ones. It seems to be this commodification of spirituality that turns out to be a strong driver for people to buy certain ritual objects. Just as in New Age, Neopaganistic religions, such as Wicca and contemporary witchcraft that also formed and expanded in the 20th century, are known to tap into the capitalist mindset of Europeans and Euro-Americans, where several objects and artefacts are sold to answer to the demand for spirituality [60]. 

Interviewees also mentioned that white sage smells better, so a preference for white sage could be caused by its volatile aromatic compounds, giving it a biological interpretation. The chemical composition of essential oils present in *Salvia* species is, among other factors, influenced by abiotic variables such as temperature and precipitation [61,62]. This might be an additional driver for people in the Netherlands, where the weather is generally cooler and wetter than in Mediterranean and Californian regions, to avoid the native flora and look for commercialized species coming from warmer and dryer conditions for its use in smudging.

The appropriation of non-Western cultural elements is often seen in a negative light, especially when these tendencies are commercialized and cause adverse impacts on the culture these elements are originally associated with [23]. Commercialized smudge sticks containing North American species are not always labeled as Native American ritual ingredients and Dutch consumers seem generally unaware of the potential negative ecological and social impact on Native American livelihoods, including cultural health and traditions. The availability of smudge sticks in the Netherlands may also have inspired people to transform and personalize smudge sticks by creating them with native or cultivated plants, in a sense reminding the Dutch population that plant smoke can be used for cleansing and protection (see Section 3.3). However, to guarantee the sustainable availability of smudge stick species, scientific research is needed to study the potential impacts of commercial harvesting with the aim of providing a guideline for harvest control and conservation plans.

## 4. Materials and Methods

### 4.1. Collecting Specimens

We collected smudge sticks, loose leaves for burning (only species that were also seen in smudge sticks to aid with identification), and woody material sold to use as incense between September and November 2021, in March 2023 and in May–June 2024 from online and physical shops. Online shops directed to the Dutch market were found through Google queries using the keywords: “smudge sticks” combined with the Dutch terms for ordering (“bestellen”) and buying (“kopen”). We visited physical New Age shops in the cities Amsterdam, Utrecht, and Wageningen and attempted to obtain as many different plant species as possible. 

Species present in smudge sticks were identified using the Naturalis Biodiversity Center herbarium (L) and online scans from Kew Data Portal (https://data.kew.org/). Furthermore, for identifying North American species in the Asteraceae family we consulted the Flora of North America (http://floranorthamerica.org), to identify species in the *Salvia* genus and *Eriodictyon* we used [63,64,65,66]. For the identification of a possibly horticultural *Lavandula* we consulted KeyBase (https://keybase.rbg.vic.gov.au/keys/show/7633, accessed 6 August 2024). Woody material was identified by a wood anatomy expert at Naturalis. We checked the current scientific names by consulting The World Flora Online (https://www.worldfloraonline.org/). To aid with plant identification, wholesalers were contacted and asked where certain plant material originated from. Plant species’ distributions were found in https://plants.usda.gov and in https://powo.science.kew.org (both accessed 6 June 2024). 

Voucher specimens were deposited at the herbarium of Naturalis Biodiversity Center (L) in Leiden, the Netherlands. When species could be identified without disassembling the smudge sticks, we deposited the intact sticks in the Economic Botany collection of the same institute. For the figures, we photographed smudge sticks in front of a black velvet canvas and created a collage using PowerPoint.

### 4.2. Interviews

Semi-structured interviews were held in the same fieldwork periods as above with 11 persons, of which four vendors of smudge sticks, five persons working in the herbal industry (e.g., phytotherapists and organizers of herbal medicine workshops) and two distributors of self-made smudge sticks. Participants were recruited through the first author’s contacts, further snowball sampling and Google searches. Most informants were interviewed by phone, while two were visited in-person. After introducing our research and obtaining permission to use their interview data in our research, we asked them the following questions: What plant species are included in smudge sticks? What is the use of each species in a smudge stick? Do you or people that you know prefer white sage (*Salvia apiana* Jeps.) instead of native Dutch species (e.g., *Artemisia vulgaris* L.) and why? We made sure that when we asked about white sage the interviewees knew we were specifically interested in *S. apiana* (as opposed to other species that look like it and are confused with it such as *S. officinalis*) by discussing its morphological characteristics and its origin. Interviews were conducted in Dutch, following the ethical guidelines of the International Society for Ethnobiology [67]. 

### 4.3. Questionnaires 

Apart from the in-depth semi-structured interviews, we used data from 33 questionnaires that were distributed and filled in during the witches fair “Hexfest” in Oss, the Netherlands, in February 2020. These questionnaires were used for another project to gain insights into plant species and their uses, plant origins (i.e., wild-picked, garden-grown or bought), and origin of knowledge of plant use (i.e., family, books, internet), among people that self-identify as witches. In these questionnaires people were also asked to freelist plants that they used for medicinal, ritual, and/or religious purposes. The specific data collected regarding plants used for smudging were used in our analysis. 

## 5. Conclusions

Smudge sticks in the Netherlands are found in shops that specialize in esoteric products. These dried plant bundles are often made up of different North American species, although mainly *Salvia apiana*. The current demand for smudge sticks possibly has a negative impact on local plant species’ populations and on essential cultural practices of local Native American livelihoods. An assessment of the smudge sticks species’ population status and trends is recommended, as well as an inventory of the commercial supply and demand of their herbal products. Also, considering that more steps in the chain of smudge stick commercialization are presumably observed in North America, a collaboration between ethnobotanists on both the European and North American continents, could prove fruitful to give further insights into plant conservation necessities regarding smudge stick plant species. 

As capitalist-oriented New Age and Neopagan movements, such as Wicca, modern Witchcraft, and others, attain a growing number of followers, it will be necessary to keep track of their ethnobotanical journey. What drives people in these movements in their choice of ritual plants? How will plant preferences change over time? And how will these preferences impact species’ populations in the wild? These movements are relatively new, but do not stand on their own, as they assimilate and adapt rituals and ceremonies from all over the globe [9,68], including plant usage as we have shown. Smudge sticks are only one example of ritualistic commodified objects containing plant material that were assimilated. Resins are another example, but they are challenging to identify, especially when shops are not transparent about the origin of their products. Other plant species, that are not burned, but, for example, rather used as decoration or ingested (e.g., “cacao ceremonies”) in New Age and Neopaganistic ceremonies are still open for investigation. Further research might elucidate if people intrinsically associate smells with protective properties, but “forgot” traditional native ritual plants making it possible for the exotic smudge sticks to fill this ethnobotanical niche. 

## Figures and Tables

**Table 1:** Species found in our collected smudge sticks sourced from online and physical shops in the Netherlands.

Family	Species/Collection ^1^	Names Product Label ^2^	Species Distribution Range
Anacardiaceae	indet. ^3^ (not *Pistacia lentiscus*)/IPG317	Pistacia lentiscus	-
Asteraceae	*Artemisia* subg. Tridentatae/BZ1, BZ3, BZ15, IPG315	Mountain sage (BZ1, BZ3), wee sage (BZ3), shasta sage (IPG315), blue sage (IPG315) (English), blauwe salie (BZ15), woestijnsalie (BZ3) (Dutch)	Western USA
Asteraceae	*Artemisia californica* Less./BZ13	Desert sage (English)	California (USA) and Baja California (Mexico)
Asteraceae	*Artemisia ludoviciana* Nutt./BZ5, IPG183	Dakota witte salie (Dutch)	Canada, USA, and Mexico
Asteraceae	*Artemisia tridentata* Nutt./BZ10	Blue sage, big sagebrush (English)	Western Canada to Baja California (Mexico)
Asteraceae	*Artemisia vulgaris* L./BZ17, BZ19, BZ22, BZ25	Bijvoet, zwarte salie (Dutch), Mugwort (English)	Temperate Eurasia, introduced in Canada and USA
Asteraceae	*Matricaria chamomilla* L./BZ24	Kamille (Dutch)	Eurasia, introduced in Canada and USA
Asteraceae	*cf. Pseudognaphalium* sp. 1 (including colored specimens)/IPG318, IPG319, IPG320, IPG314	Verbascum	-
Asteraceae	*cf. Pseudognaphalium* sp. 2/IPG313, IPG331	Groene salie (Dutch)	-
Boraginaceae	*Eriodictyon californicum* (Hook. & Arn.) Decne. or *Eriodictyon trichocalyx*/BZ9	Yerba Santa (Spanish)	Western USA (Oregon and California)
Boraginaceae	*Eriodictyon angustifolium* or *Eriodictyon altissimum*/BZ16	Yerba Santa (Spanish)	Western USA
Boraginaceae	*Eriodictyon* sp./IPG319	Yerba Santa (Spanish)	Western USA
Burseraceae	*Bursera graveolens* Triana & Planch/IPG316	Palo Santo (Spanish), holy wood (English)	Mexico to northwestern Venezuela and Peru
Cupressaceae	*Calocedrus decurrens* (Torr.) Florin/BZ2	Pine (Dutch)	Western USA (Oregon and California) and Mexico (Baja California)
Cupressaceae	*Thuja* sp./BZ6	Cedar sage (English)	USA and Canada, introduced in Europe
Hypericaceae	*Hypericum perforatum* L./BZ18	Sint-Janskruid (Dutch)	Eurasia, introduced in USA and Canada
Lamiaceae	*Lavandula dentata* L./SS2024-5	Lavendel (Dutch)	Western Mediterranean region and north-eastern Africa
Lamiaceae	*Salvia apiana* Jeps./BZ4, BZ8, BZ11, BZ12, BZ14, IPG318, IPG317, IPG320, IPG324, IPG321	Witte salie, Californische witte salie, salie (Dutch), White sage, White Californian Sage (English), Dragon’s blood salie (name given to a red colored smudge stick; English and Dutch)	Western USA (California) and northwestern Mexico
Lamiaceae	*Salvia fruticosa* Mill./IPG323	Griekse salie (Dutch), Greek ceremonial sage (English)	Eastern Mediterranean region
Lamiaceae	*Salvia officinalis* L./BZ20, BZ23, BZ26, IPG322	Salie (Dutch), smudge salie (both)	Europe and cultivated worldwide
Lamiaceae	*Salvia rosmarinus* Schleid./IPG322	Rozemarijn (Dutch)	Europe and cultivated worldwide
Myrtaceae	*Eucalyptus* sp. (colored (claiming with red dracaena dye) and uncolored)/IPG325, IPG318	Eucalyptus & Dragon’s blood (English)	Australia and cultivated worldwide
Plumbaginaceae	*Limonium sinuatum* Mill./IPG320, IPG319	Sinuata, Statice sinuata (Latin/Scientific name)	Mediterranean region, introduced in western USA and Mexico
Poaceae	indet./BZ7	Sweet grass (English)	-
Poaceae	*Phalaris* sp./IPG317	Phalaris (Latin/Scientific name)	-
Poaceae	indet. (painted blue)/IPG320	- ^4^	-
Rosaceae	*Rosa* sp./BZ21	Rozen (Dutch)	Northern Hemisphere and cultivated
Rosaceae	*Rosa* sp. (colored petals)/IPG324	Chakra blaadjes (Dutch)	Northern Hemisphere and cultivated
Verbenaceae	*Verbena bonariensis* L./BZ27	IJzerhart [sic] (Dutch)	South America and cultivated elsewhere
Indet.	indet. (painted blue)/IPG318	- ^4^	-

^1^ Voucher specimens of collected smudge sticks were deposited at the herbarium of Naturalis Biodiversity Center (L) in Leiden, the Netherlands. ^2^ Vernacular names (local and “scientific”) used in trade which do not always reflect official vernacular names as used in floras. ^3^ Indeterminate species. ^4^ Species is not listed on store label.

## Data Availability

The original contributions presented in the study are included in the result section of the article, further inquiries can be directed to the corresponding author.
